# Mapping of Quantitative Trait Locus (QTLs) that Contribute to Germination and Early Seedling Drought Tolerance in the Interspecific Cross *Setaria italica*×*Setaria viridis*

**DOI:** 10.1371/journal.pone.0101868

**Published:** 2014-07-17

**Authors:** Lufeng Qie, Guanqing Jia, Wenying Zhang, James Schnable, Zhonglin Shang, Wei Li, Binhui Liu, Mingzhe Li, Yang Chai, Hui Zhi, Xianmin Diao

**Affiliations:** 1 Institute of Crop Sciences, Chinese Academy of Agricultural Sciences, Beijing, China; 2 Institute of Millet Crops, Hebei Academy of Agricultural and Forestry Sciences, Shijiazhuang, China; 3 Institute of Dry Land Agriculture, Hebei Academy of Agricultural and Forestry Sciences, Hengshui, China; 4 College of Life Sciences, Hebei Normal University, Shijiazhuang, China; CIRAD, France

## Abstract

Drought tolerance is an important breeding target for enhancing the yields of grain crop species in arid and semi-arid regions of the world. Two species of *Setaria*, domesticated foxtail millet (*S. italica*) and its wild ancestor green foxtail (*S. viridis*) are becoming widely adopted as models for functional genomics studies in the Panicoid grasses. In this study, the genomic regions controlling germination and early seedling drought tolerance in Setaria were identified using 190 F_7_ lines derived from a cross between Yugu1, a *S. italica* cultivar developed in China, and a wild *S. viridis* genotype collected from Uzbekistan. Quantitative trait loci were identified which contribute to a number of traits including promptness index, radical root length, coleoptile length and lateral root number at germinating stage and seedling survival rate was characterized by the ability of desiccated seedlings to revive after rehydration. A genetic map with 128 SSR markers which spans 1293.9 cM with an average of 14 markers per linkage group of the 9 linkage groups was constructed. A total of eighteen QTLs were detected which included nine that explained over 10% of the phenotypic variance for a given trait. Both the wild green foxtail genotype and the foxtail millet cultivar contributed the favorite alleles for traits detected in this trial, indicating that wild *Setaria viridis* populations may serve as a reservoir for novel stress tolerance alleles which could be employed in foxtail millet breeding.

## Introduction

Droughts reduce the water available in the soil to crop plants, producing stress and decreasing grain production [1]. Global climate change, combined with increased urban demand for freshwater, means that agricultural production will be increasingly constrained by water supply while demand for food continues to grow [2]. In order to avoid catastrophic yield losses and to meet the food requirement of the rapidly growing world population it will be necessary to develop new crop varieties which can produce more grain while using less water. Previous genetic investigations have shown that drought tolerance is a complex trait controlled by multiple small effect QTLs (quantitative trait loci), and increasing of the efficiency of water use always certainly involves trade-offs with growth [3]–[4]. QTLs for drought tolerance have been identified for several major crop species including rice [5]–[6], maize [7], wheat [8], barley [9], sorghum [10], pearl millet [11] and soybean [12]. However, to date, no published study has examined the genetic architecture of drought tolerance in Setaria.

Foxtail millet (*Setaria italica*) and its wild relative green foxtail (*S.viridis*) are rapidly becoming a novel model for deciphering abiotic stress and C_4_ photosynthesis biology as a result of the small genome size, self-fertilization, short growing cycle, small growth stature and efficient genetic transformation of the species [13]–[16]. Foxtail millet is more drought tolerant than many grain crops and has been long used in dryland agricultural production across the arid and semi-arid regions of Northern China. Foxtail millet seeds were found to require only 26% of their seed weight in water for successful germination, while other cereals require at least 45% of their seed weight [17]. The water use efficiency (WUE) of foxtail millet, a measure of how much water is required to produce one unit of biomass, is also higher than other grain crops. Maize requires 470 grams of water to produce 1 gram of dry biomass, wheat requires 510 grams of water, and foxtail millet requires a modest 257 grams [17]. Several morphological and physiological adaptations have been reported to be associated with drought tolerance and higher WUE in foxtail millet, including dense and deep root systems, smaller leaf area, and thickening of the cell walls [18].

A high throughput assessment and screening of 17,313 accessions of foxtail millet genotypes for drought tolerant from China was performed by measuring seedling survival rates under multiple drought stress treatments. More than two hundred lines were classified into the highest category of drought tolerance (grade 1), including the cultivar “Yugu1” [18]. A high throughout screen for drought tolerance in foxtail millet during germination was developed which used polyethylene glycol (PEG-6000) to generate simulated water shortage environments [19]. Multiple methods of gene expression profiling have been employed to identify genes related to drought tolerance in foxtail millet, and these methods identified hundreds of significant changes in expression that corresponded to genes involved in metabolism, proteolysis, and signaling [20]–[23]. However, the detailed genetic mechanisms responsible for variation in drought tolerance among foxtail millet lines remain uncharacterized.

In this study a set of osmotic stress-related QTLs were identified in population generated from an interspecific cross between domesticated foxtail millet and the wild green foxtail of Setaria species. QTLs for seed germination and early seedling development were characterized using a set of SSR markers, and the markers identified in this study as genetically linked to loci conveying increased drought tolerance can be used in the future for marker assisted selection (MAS) approaches to developing even more drought tolerant foxtail millet cultivars.

## Materials and Methods

### 1. Plant material

The two parental lines used to generate the interspecific mapping population were the *S.italica* cultivar “Yugu1” (whose genome sequence has been released by Bennetzen et al. [24]) and the *S. viridis* accession “W53” (collected from Uzbekistan). Hybridization between parents was performed using the protocol described in Wang et al. [25]. *S. italica* was used as the female parent, F_1_ hybrid plants were identified using dominant morphological markers from *S. viridis*, and later verified using SSRs. A single validated F_1_ plant was self-pollinated to produce an F_2_ population, consisting of 190 plants. Each F_2_ plant was used to generate a single recombinant inbred line (RIL) (F_7_ generation) through single seed descent (SSD) for the use in this trial.

### 2. SSRs genotyping and genetic linkage map construction

Template DNA was extracted from leaves of the sampled Setaria accessions using the CTAB method [26]. SSR primers sourced from previous studies [27]–[28] and those developed for this project were used for PCR genotyping reactions. PCRs were performed in 10 µl volumes containing 50ng of genomic DNA, 200 µm dNTPs, 1 µl 10×PCR buffer, 0.5 µm each of forward and reverse primer, 0.75 U Taq polymerase (Takara). The PCR profile included an initial denaturation step at 94°C, for 4 min, followed by 35 cycles of 40 s at 94°C, 40 s at annealing temperature (45–60°C) and 1 min at 72°C. A final extension step of 72°C for 5 min completed the program. 10 mM EDTA, bromophenol blue and xylene cyanol were added to each reaction. Samples were denatured at 94°C for 5 min, and cooled for 5 min on ice. A total of 5 µl of each sample was subjected to electrophoresis at 80 w on 5% denaturing polyacrylamide gels for 40 min. After electrophoresis, gels were silver-stained as described in Bassam et al. [29]. The SSRs with clear and scorable amplification were selected for further diversity study and linkage map construction.

SSRs that were polymorphic between the two parents of the mapping population were used to genotype the 190 F_7_ lines. The distorted segregation of SSR markers were identified by chi-square test to determine if they fitted to the expected Mendelian segregation ratio. The linkage map was constructed using MAPMAKER version 3.0 [30]. A LOD score of 3.0 and a default distance threshold of 80 Haldane cM of MAPMAKER were set to identify linkage groups. The Kosambi mapping function was used to convert recombination frequencies into map distances [31].

### 3. Traits evaluations

Mature seeds of each RIL were surface sterilized by soaking in 75% alcohol for 5 min, followed by 3 washes in deionized and distilled water (ddH_2_O). For each line, 50 seeds were germinated on filter paper in each petri-dish under dark conditions and a constant temperature of 28 (±1)°C. For osmotic stress conditions, 5 mL of a 20% w/v PEG6000/water solution were added into each petri-dish while in control conditions 5 mL ddH_2_O were added. For each genotype in each condition (stress or control) three independent biological replicates of 1 petri-dish were scored. The water potential of the germination medium was approximately −0.5 MPa. For eight days, the number of seeds in each petri-dish which had germinated was recorded. A seed was considered germinated when both plumule and radical had emerged to at least 5 mm long. Total germination rate for each line under osmotic stress conditions was calculated as (number of seeds germinated under osmotic stress conditions)/(number of seeds germinated under control conditions). For radical root length (RL), coleoptile length (CL) and lateral root number (LRN) measurements, 20 seeds of each line were germinated under 16/8 of light/dark environment for 6 days, and 5 seedlings were selected randomly for evaluations, and three repeats were carried out. Parameters were defined as follows:

PI = nd_2_×1+nd_4_×0.75+nd_6_×0.5+nd_8_×0.25 [32]

Promptness index (PI) is the percentage of seed which had germinated at 2^nd^, 4^th^, 6^th^ and 8^th^ day of observation as indicated by nd_2_, nd_4_, nd_6_ and nd_8_. Percentage of germination stress tolerance index (GSI) is determined as below:

GSI (%) =  (PI of stressed seeds/PI of control seeds) ×100

RL, CL and LRN were measured and recorded 6 days after germination was observed for that seedling both under control and stress conditions, and further denoted as, RLC and RLS, CLC and CLS, and LRNC and LRNC, respectively. Drought related parameters were defined as follow:

Radical length decrease (RLD) =  RLC — RLS

Coleoptile length decrease (CLD) =  CLC — CLS

Lateral root number decrease (LRND) =  LRNC — LRNS

Radical length relative (RLR) =  RLS/RLC

Coleoptile length relative (CLR) =  CLS/CLC

Lateral root number relative (LRNR) =  LRNS/LRNC

Radical length index (RLI) =  RLD/RLC

Coleoptile length index (CLI) =  CLD/CLC

Lateral root number index (LRNI) =  LRND/LRNC

Seedling survival rate (SR) was assessed using plants grown in potting soil under greenhouse conditions at a constant temperature of 28°C under 16h/8h of light/dark environment. For each line, three replicates (plots) were grown in a randomized arrangement. Seeds were planted in soil that had been watered to saturation. After seedlings emerged from the soil, hand thinning was carried out for each line so that each pot contained 10 seedlings. All water was withheld from a given line once the third leaf emerged and continued until the soil moisture content was reduced to 5±2%, and the leaves of the seedlings had dried to withering. Plants were then rewatered to soil saturation, and seedlings showing fresh green leaves within 72 hours were considered to have survived under drought stress and used to calculate the survival rate of each line. Heritability of direct traits including germinating rate at 2^nd^, 4^th^, 6^th^ and 8^th^ day, radical root length (RL), coleoptile length (CL) and lateral root number (LRN) were estimated from the variance between replicates using ANOVA analysis calculated by SPSS (IBM Corporation, 2009).

### 4. QTL mapping

Each trait was analyzed in the trial using composite interval mapping (CIM) and the standard model (Model 6) as implemented by Windows QTL Cartographer V2.5 [33], with a walk speed of 1.0 cM and a window size of 10.0 cM. Significance for QTL detection was firstly determined using permutation tests (significance level  = 0.05, 1.000 permutations) and then a significant LOD score of 2.5 in average for each trait was used as threshold in this study. The QTL region was identified as a 1-LOD drop-off interval from the peak of a significant QTL.

## Results

### 1. Phenotyping and heritability

**Figure 1:**
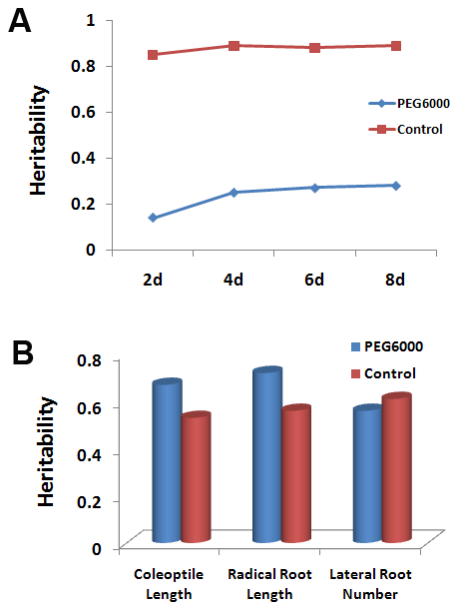
Heritability of germination (A) and morphological (B) traits measured under both normal and PEG6000 osmotic conditions.

The *S. italica X S. viridis*, SI×SV, RIL population showed high variability in both seed germination and seedling surviving rate phenotypes (**Table 1**). Promptness index (PI), Radical root length (RL), Coleoptile length (CL) and Lateral root number (LRN) were significantly inhibited under PEG6000 osmotic conditions. The same trend was also seen for Survival rate (SR). RL and LRN showed the largest changes between control and osmotic stress conditions. Heritability of germination traits decreased under osmotic stress conditions, whereas, heritability of morphological traits was stable (**Figure 1**).

**Table 1:** Seed germination and seedling related trait values for *S.italica* and *S.viridis* parents and recombinant inbred lines (RILs) under diverse treatments.

Traits			*S.italica*	*S.viridis*	RIL mean	RIL range
Promptness index	Control	PI_C	2.05	2.48	2.22±0.34	0.37∼2.50
	PEG6000 Osmotic	PI_S	0.85	0.73	1.16±0.32	0.05∼1.91
Radical root length (mm)	Control	RL_C	9.00	7.60	10.01±1.58	5.70∼15.30
	PEG6000 Osmotic	RL_S	0.60	1.50	1.29±0.59	0.00∼4.40
Coleoptile length (mm)	Control	CL_C	3.00	3.00	2.57±0.73	0.40∼4.50
	PEG6000 Osmotic	CL_S	0.70	0.50	0.53±0.24	0.00∼1.20
Lateral root number	Control	LRN_C	9	4	12.1±5.2	0∼26
	PEG6000 Osmotic	LRN_S	2	2	0.9±1.0	0∼7
Germination stress tolerance index		GSI_D	0.41	0.29	0.52±0.12	0.09∼0.79
Survival rate		SR_D	0.65	0.90	0.61±0.16	0.08∼0.93
Radical root length decrease (mm)		RLD_D	8.40	6.10	8.70±1.64	4.80∼14.30
Radical root length relative		RLR_D	0.07	0.20	0.13±0.06	0.00∼0.43
Radical root length index		RLI_D	0.93	0.80	0.87±0.06	0.57∼1.00
Coleoptile length decrease (mm)		CLD_D	2.30	2.50	2.00±0.68	0.40∼3.80
Coleoptile length relative		CLR_D	0.23	0.17	0.21±0.10	0.00∼0.64
Coleoptile length index		CLI_D	0.77	0.83	0.79±0.10	0.36∼1.00
Lateral root number decrease		LRND_D	7	2	11.0±5.2	0∼26
Lateral root number relative		LRNR_D	0.22	0.50	0.09±0.13	0∼1
Lateral root number index		LRNI_D	0.78	0.50	0.91±0.13	0∼1

### 2. Genetic map construction

**Figure 2:**
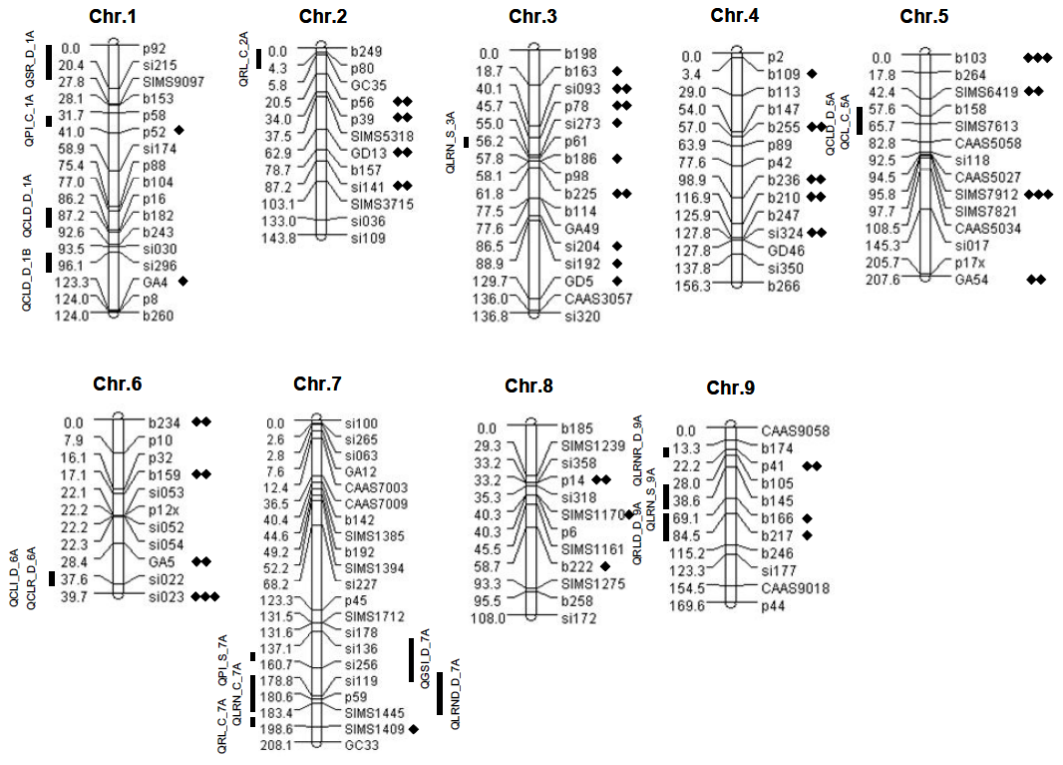
Genetic linkage map constructed using 128 SSR markers. Black lines represent QTLs detected in this trial; Solid rhombuses represent skewed markers (Regions) detected in this population and two other recombinant inbred line populations (**see Table S2**), number of rhombuses means number of populations identified segregating deviation in target regions.

A linkage map of the recombinant inbred lines (RIL) population derived from *S.italica* and *S. viridis* was constructed using 128 SSR markers and spans 1293.9 cM across 9 linkage groups with an average of 14 markers per linkage group (**Figure 2**). The average interval between consecutive markers is 10.2 cM, ranging from 0.0 cM between co-segregating markers to the largest gap on chromosome 5 between markers si017 and p17x. Another gap was identified on chromosome 7 between markers of si227 and p45. All SSRs on the genetic map were aligned with the corresponding physical location (**Table S1**) of the marker sequences in the *S.italica* (Yugu1) genome assembly [24].

A number of markers genotyped in the RIL population showed genotype frequencies that significantly varied from the expected 1∶1 ratio between the parental genotypes (**Table S2,** **Figure 2**). A total of 35 SSRs (27.3%) showed segregation distortion, of which, 28 (80%) deviated towards the cultivated species, Yugu1. Twenty-one skewed SSRs overlapped with regions also reported to exhibit segregation distortion in a second interspecific recombinant *Setaria* population (B100×A10) [24]. Three of the SSRs were located in genome regions which exhibited segregation distortion in a third intraspecific recombinant *S. italica* population, (A2×Zhang gu) [34].

### 3. Identification of QTLs

#### QTLs detected under normal conditions

A total of 5 QTLs (LOD>2.5) were identified in the SI × SV population under control conditions (**Table 2** **,** **Figure 2** **and Figure S1**). For CL, one QTL (*QCL_C_5A*), which explained 11% of the phenotypic variance, was identified on chromosome 5. Two QTLs (*QRL_C_7A* and *QRL_C_2A*) explaining 7% and 10% of the phenotypic variance of RL were detected on chromosome 7 and 2. In terms of LRN, one QTL (*QLRN_C_7A*) contributing 10% of the total variance was characterized on chromosome 7. One QTL (*QPI_C_1A*) explaining 7% the phenotypic variance of PI was identified on chromosome 1. *S. viridis* contributed the positive (increased value) alleles for 2 loci (*QCL_C_5A* and *QRL_C_2A*) under normal conditions. *S. italica* contributed the favorable allele for the remaining loci.

**Table 2:** Significant QTLs detected in RIL populations.

Category	QTL	Trait	Chromosome	Peak (cM)	Peak LOD	Left boundary (cM)	Right boundary (cM)	*A* ^a^	*R^2^*
Control	QCL_C_5A	CL_C	5	66.1	4.45	52.0	76.0	−0.25	0.11
	QRL_C_7A	RL_C	7	196.3	2.51	195.2	197.4	0.42	0.07
	QRL_C_2A	RL_C	2	11.0	4.18	0.8	19.9	−0.52	0.10
	QLRN_C_7A	LRN_C	7	179.4	4.44	170.0	187.5	1.63	0.10
	QPI_C_1A	PI_C	1	39.9	2.93	36.1	40.3	0.11	0.07
PEG6000 Osmotic	QLRN_S_3A	LRN_S	3	55.2	2.65	53.9	56.4	−0.30	0.06
	QLRN_S_9A	LRN_S	9	65.1	3.27	49.1	75.6	−0.41	0.12
	QPI_S_7A	PI_S	7	150.8	2.74	143.8	157.5	0.10	0.09
Drought tolerance-related	QGSI_D_7A	GSI_D	7	154.1	3.91	141.2	174.1	0.05	0.14
	QCLD_D_1A	CLD_D	1	85.5	3.12	79.6	86.9	0.18	0.07
	QCLD_D_1B	CLD_D	1	100.8	2.95	95.7	109.2	0.22	0.10
	QCLD_D_5A	CLD_D	5	63.9	3.47	54.0	74.1	−0.20	0.09
	QCLR_D_6A	CLR_D	6	37.7	2.71	36.3	38.2	0.02	0.06
	QCLI_D_6A	CLI_D	6	37.7	2.71	36.3	38.2	−0.02	0.06
	QRLD_D_9A	RLD_D	9	89.6	3.16	78.7	100.6	−0.58	0.12
	QLRND_D_7A	LRND_D	7	179.0	4.32	170.3	186.7	1.65	0.10
	QLRNR_D_9A	LRNR_D	9	26.3	2.77	25.2	26.7	−0.14	0.06
	QSR_D_1A	SR_D	1	10.4	3.99	0.2	19.8	0.61	0.13

a: Positive values means favorite alleles were contributed by female parent Yugu1; Negative values means favorite alleles were contributed by male parent W53.

#### QTLs detected under osmotic stress conditions

A total of 3 QTLs were detected under PEG6000 osmotic conditions. Two QTLs (*QLRN_S_3A* and *QLRN_S_9A*) explaining 6% and 12% of the phenotypic variance of LRN were identified on chromosome 3 and 9. One QTLs (*QPI_S_7A*) contributing 9% of the phenotypic variance of PI was characterized on chromosome 7. *S. viridis* provided the favorable (increased value) alleles for 2 loci of *QLRN_S_3A* and *QLRN_S_9A* under osmotic conditions. *S. italica* contributed the favorable allele to *QPI_S_7A*.

#### Drought tolerance-related QTLs

Ten QTLs responsible for variation in drought tolerance among the lines of the SI × SV population were identified. One QTL (*QGSI_D_7A*) explaining 14% of the phenotypic variance of GSI was detected on chromosome 7. For CLD, three QTLs (*QCLD_D_1A*, *QCLD_D_1B* and *QCLD_D_5A*) contributing 7%, 10% and 9% of the phenotypic variance were identified on chromosomes 1 and 5. One QTL (*QCLR_D_6A*) explaining 6% of the phenotypic variance of CLR was detected on chromosome 6. As to CLI, one QTL (*QCLI_D_6A*) contributing 6% of the total phenotypic variance was identified on chromosome 6. In terms of RLD, one QTL (*QRLD_D_9A*) explaining 12% of the phenotypic variance was identified on chromosome 9. For LRND, one QTL (*QLRND_D_7A*) contributing 10% of the phenotypic variance was characterized on chromosome 7. One QTL (*QLRNR_D_9A*) explaining 6% of the phenotypic variance of LRNR was detected on chromosome 9. As to SR, one QTL (*QSR_D_1A*) contributing 13% of the phenotypic variance was characterized on chromosome 1. *S. viridis* provided the favorable (increased value) alleles for 4 loci (*QCLD_D_5A*, *QCLI_D_6A*, *QRLD_D_9A* and *QLRNR_D_9A*) for drought tolerance. *S. italica* contributed the favorable allele to *QGSI_D_7A*, *QCLD_D_1A*, *QCLD_D_1B*, *QCLR_D_6A*, *QLRND_D_7A* and *QSR_D_1A*.

## Discussion

### 1. Response of a SI × SV interspecific cross population to drought stress

PEG has been widely used as a proxy for drought stress in evaluations of drought tolerance in crop species, which could be used to simulate water shortage conditions precisely by variant concentrations of osmotic solutions [32], [35]. Indicators measured at seedling stage always represent surrogates for drought response in foxtail millet under real field conditions [18]. In this trial, five characters including PI, RL, CL, LRN and SR were measured in an interspecific recombinant population under control and osmotic stress conditions. All five traits showed significant variation in response to water availability. The traits RL and LRN showed higher sensitivity to drought conditions, consistent with previous reports [36]. Coleoptile length is considered to play a vital role in the success of deep sown wheat [37] and barley [38] in semi-arid environments. A similar trend of decreased coleoptile length under osmotic stress conditions was observed in our study of Setaria. PI and SR were argued to be essential methods of assessing drought tolerance [35], a view that is supported by the results of this project. Measurements of PI and SR could be possibly used as direct proxies for yield under drought stress in cultivar improvement programs aimed at developing higher yield *Setaria* varieties for arid or semi-arid areas of the world.

Heritability analysis conducted in this trial showed that the germination rate (**Figure 1A**) of Setaria is affected by osmotic environment, the observed heritability of this trait is lower than that reported in rapeseed [39], tomato [40] and wheat [41]. As a result, a longer or more intensive selection program would be required to develop varieties of Setaria which exhibit high germination under drought conditions.

### 2. Genetic linkage map and distorted segregation of SSRs

At least two interspecific mapping populations have been reported between *Setaria italica* and *Setaria viridis*, including Longgu25× Pagoda Flower Green [25] and B100×A10 [24]. Meanwhile, a set of linkage maps constructed using RFLPs [25], [42], InDels [27], [34] and SNPs [24], [43] have been utilized for QTLs mapping and map-based gene cloning analysis in this target species. In the present study, an interspecific RIL population and one novel genetic linkage map including 128 SSRs were built in *Setaria*. Owing to the increasing number of SSRs compared with previous studies [27], markers were roughly evenly distributed across the nine chromosomes of foxtail millet, with an average of 14 markers per linkage group. Large scale genomic rearrangement (including inversions or translocations) which might result in hybrid fertility issues were not observed in the cross between Yugu1 (*S.italica*) and W53 (*S.viridis*), which was consistent with previously published studies [24]–[25] on interspecific and intraspecific recombinant populations, although the comparison of the genetic map constructed in this study to previous one [27] did reveal several small segmental inversions and rearrangements on chromosome 1, 3, 4 and 9 (Figure S2). This result, and the similarity of the degree of chromosomal pairing as judged by the total genetic map lengths of diverse populations, is consistent with the suggestion of Harlan and deWet [44] that *S.italica* and *S.viridis* are taxonomically the same species and should be considered as subspecies. Recent analysis of microsatellite transferability between *S.italica* and *S.viridis* also support the same conjecture [45].

Deviations from Mendelian segregation ratios may be due to selection at pre-zygotic or post-zygotic stages. During hybridization between genetically distant parents (e.g. cultivar and wild relatives), the alleles of wild species could be frequently lost, which would lead to severe distorted segregation [46]. In this study, quite a few SSRs included in the linkage map showed significant deviation from the expected 1∶1 Mendelian ratio. Of the markers exhibiting segregation distortion, 80% deviated in the direction of higher abundance of Yugu1 alleles (*S.italica*). Similar biases were observed in related analysis [27], [43], [47]. These results suggest that many alleles from the wild parent *S.viridis* are lost in recombinant mapping populations, resulting in the considerably distorted segregation ratios of SSR markers observed in this study and others. According to the present study, higher rate of distorted segregation were observed both in interspecific and intraspecific cross populations (Table S2). This result is consistent with studies conducted in other crop species, such as rice [47]. Segregation distortion may arise from genomic background causes. Regions showing consistent segregating distortion among diverse populations of *Setaria* may also contain gametophyte or sterility related genes [48]. Further investigation into the genomic distributions of segregation distortion will be helpful for future breeding programs by making it more practical to introgress favorable alleles from wild relatives of foxtail millet, even when these hypothetical alleles are linked to regions of the genome exhibiting segregation distortion.

### 3. QTLs controlling drought-tolerant related traits in *Setaria*

The identification of traits, pathways and gene variants associated with drought tolerance is valuable for maximizing grain yield of crop species under moisture deficient environment. During the past decade, a large number of studies involving linkage mapping have been conducted in several crops to identify QTLs linked to drought tolerance [2], [4], [49]. In terms of seed germination and early seedling osmotic adjustment of crop species, many drought tolerant related QTLs have been identified in tomato [40], wheat [50], rice [6] and alfalfa [51], which suggested drought tolerance was a complex quantitative trait controlled by a large number of genes/QTLs of moderate effect size. Some QTL intervals contributing to more than one trait in this study, such as *QCL_C_5A* & *QCLD_D_5A*, *QLRN_C_7A* & *QLRND_D_7A*, *QPI_S_7A* & *QGSI_D_7A* and *QCLR_D_6A* & *QCLI_D_6A*, implied the close relationships between different drought related characters measured in this trial. In the present analysis of QTLs contributing to drought tolerance in *Setaria*, 18 QTLs were characterized. In eight cases favorable alleles originated from *S.viridis*, the wild relative of cultivated *S. italica*. Additive effects of alleles at homologous loci in two parental populations could cause transgressive segregation of target traits [52], which appeared to be common and associated with inbreeding in plant species [53]–[54] and might be key innovations in genetic architecture that favor adaptive radiation [55]–[56]. Our QTL mapping results revealed several major loci controlling drought tolerance in Setaria, which could be partial causations of why the large transgressive segregation seen in the values of target traits (**Table 1**), although this conjecture needs more work in order to be verified. Results of this study on RIL population also provide an opportunity to draw recommendations for a marker-assisted introgression program (introgressing favorable alleles from wild into cultivated Setaria) in the future, despite even if seedling response to PEG may have shown simpler inheritance, does not imply field drought response would follow the same pattern. Furthermore, intensive studies focused on field drought tolerance of foxtail millet are still essential to figure out this important issue. To sum up, our results highlight the potential value of crop-wild-relatives as a source of novel stress tolerance alleles which can contribute to the development of crop cultivars able to thrive under a wider range of environmental conditions, an increasingly important need in a world where global climate change is making weather patterns less and less predictable from one growing season to the next.

## Conclusions

This work represented a primary exploration of genomic regions controlling drought tolerance in Setaria. Quantitative trait locus (QTLs) mapping results of this work laid the foundation of gene exploring in Setaria for drought tolerance and marker-assisted breeding of foxtail millet cultivars. Furthermore, fine mapping of these candidate QTLs and identification of more regions contribute to drought tolerance in Setaria under field environments need to be conducted in the future.

## Supporting Information

Figure S1 **Plots of LOD values of QTLs identified in this trial.**
(TIF)

Figure S2 **Four segmental rearrangements between linkage groups constructed in this study (Left) and previously published genetic map (Right) (Jia et al., 2009).** Rearrangements of shared SSRs were indicated by dashed lines.
(TIF)

Table S1 **Physical positions and primers sequences of SSR markers used in this trial.**
(DOC)

Table S2 **SSR markers showing segregation distortion (P<0.01) in the Yugu1/W53 F7 RIL population and their co-localizations with segregating skewness regions annotated in other populations.**
(DOC)
