# Supplementary figures and images for: Mapping of Quantitative Trait Locus (QTLs) that Contribute to Germination and Early Seedling Drought Tolerance in the Interspecific Cross Setaria italica×Setaria viridis
Source: PLoS One. 2014 Jul 17;9(7):e101868. doi: 10.1371/journal.pone.0101868 (PMC4102488; doi:10.1371/journal.pone.0101868)

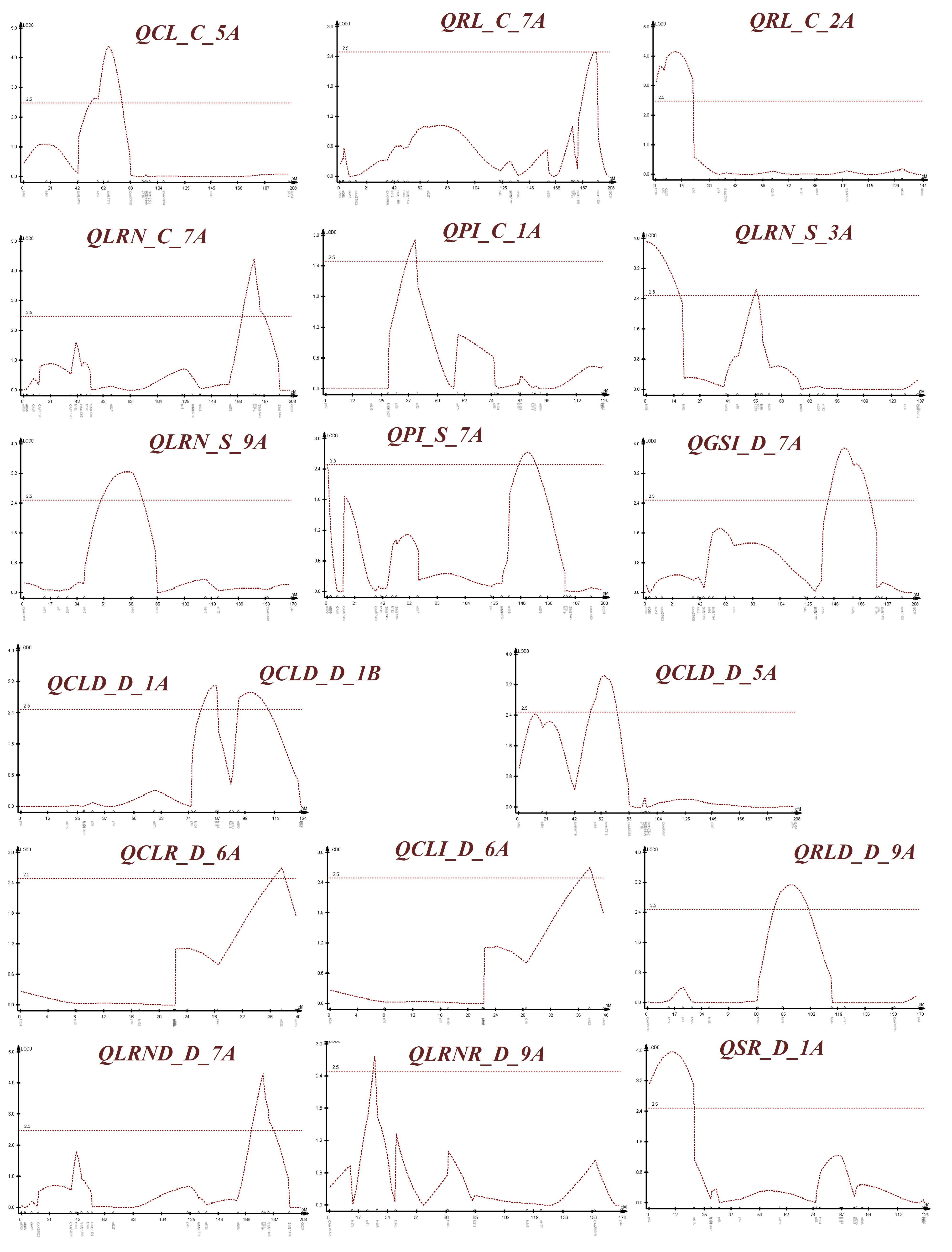

Supplement: Figure S1 — Plots of LOD values of QTLs identified in this trial. (TIF) [file pone.0101868.s001.tif]

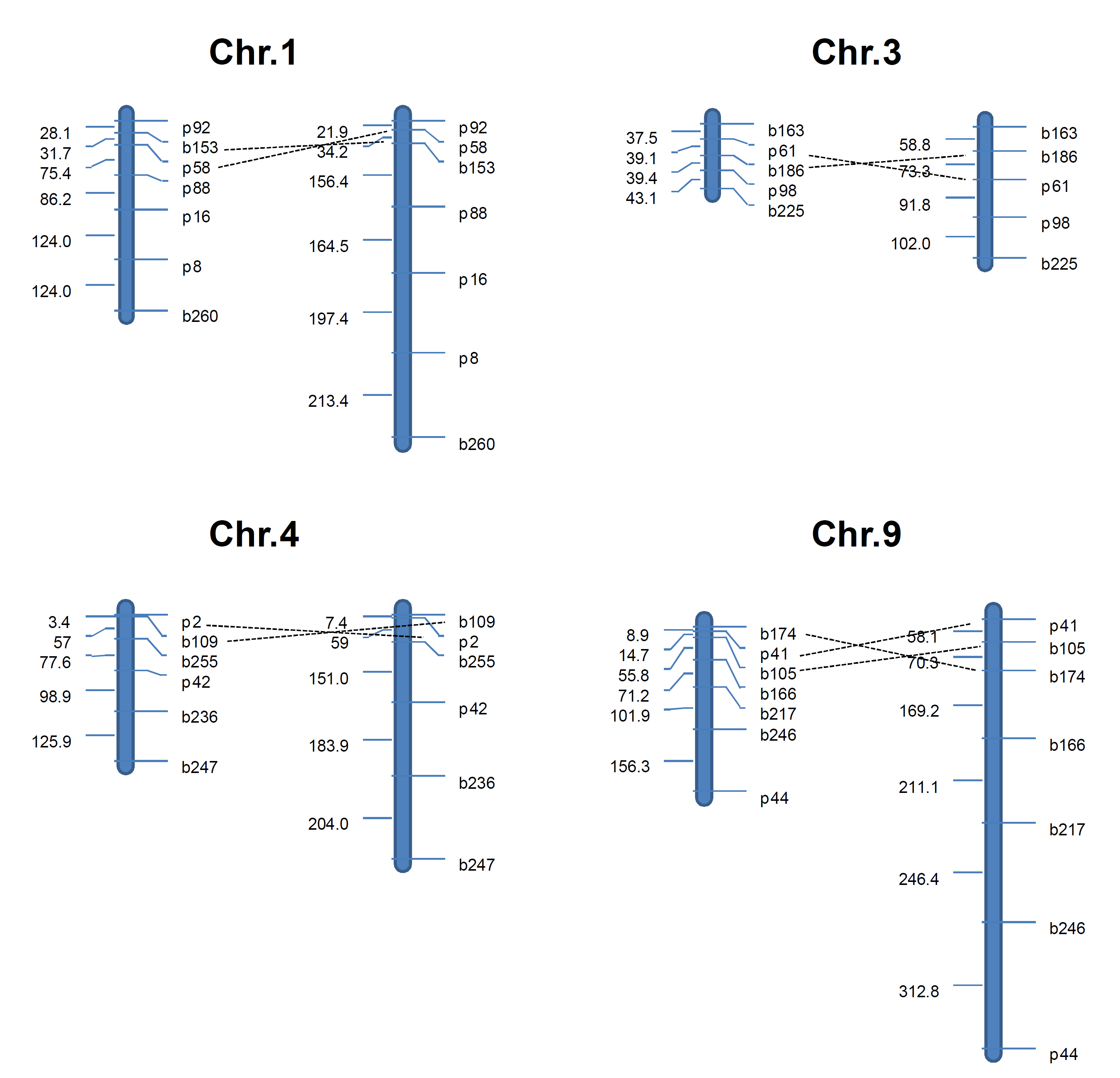

Supplement: Figure S2 — Four segmental rearrangements between linkage groups constructed in this study (Left) and previously published genetic map (Right) (Jia et al., 2009). Rearrangements of shared SSRs were indicated by dashed lines. (TIF) [file pone.0101868.s002.tif]
