# Supplementary material for: Mapping of Quantitative Trait Locus (QTLs) that Contribute to Germination and Early Seedling Drought Tolerance in the Interspecific Cross Setaria italica×Setaria viridis
Source: PLoS One. 2014 Jul 17;9(7):e101868. doi: 10.1371/journal.pone.0101868 (PMC4102488; doi:10.1371/journal.pone.0101868)
Supplement: Table S1 — Physical positions and primers sequences of SSR markers used in this trial. (DOC) [file pone.0101868.s003.doc]

| Marker Name | Chr. | Primer Sequence | Tm（℃） | Expect Size(bp) | Location(Kb) | Genetic position (cM) |
| --- | --- | --- | --- | --- | --- | --- |
| p92 | 1 | F：TGGAATTGGAACCCTTTCG  R：GCCATGCAAACAGTACCATC | 55 | 163 | 1,725 | 0.0 |
| si215 | 1 | F：GAAGGTATCCCAAAGCCTCC  R：TGACCTCGGGTTCAAGTTTC | 59 | 165 | 3,363 | 20.4 |
| SIMS9097 | 1 | F: CATCGTCCATCGATGTTCAC  R: CGTGTGCGAGAGAGAGAGTG | 60 | 221 | 3,268 | 27.8 |
| b153 | 1 | F：ACCCAACACATTCTCCTGAA  R：TGCTATCAAAATAGTGCTAGAAT | 56 | 209 | 3,363 | 28.1 |
| p58 | 1 | F：CCTGAGCTCATCCACACAAC  R：CAGCCTGGAGGAAAGGAATAG | 55 | 177 | 3,855 | 31.7 |
| p52 | 1 | F：TCACGACCGAGAACATCAAG  R：CCTGTGGTACACGATCAACG | 55 | 141 | 5,555 | 41.0 |
| si174 | 1 | F：CGACCATTTGATTTAGCGGT  R：CCTTTGGGTACAGAGGTCCA | 59 | 123 | 10,296 | 58.9 |
| p88 | 1 | F：CAAGCCACCCAGTCTAGAGG  R：TTCATCAGAACTGCGCAAAC | 57 | 199 | 29,834 | 75.4 |
| b104 | 1 | F：TGAGCTTAAAGAGTGGAAATGC  R：TGCCATCACACCCACAC | 57 | 230 | 30,697 | 77.0 |
| p16 | 1 | F：TTTCTCCCTCTCTCGATTCC  R：AAATTGGCGTGCTAACAACC | 55 | 213 | 32,947 | 86.2 |
| b182 | 1 | F：CCGATCAAATAATGCGAACA  R：TGCATCTTGCACGGATACAC | 55 | 199 | 32,831 | 87.2 |
| b243 |  | F：GATCAAGTCGGAGGCTATGG  R：ACAAGTTCTCCACCCAAGG | 55 | 167 | Unknown | 92.6 |
| si030 | 1 | F：AGTAACACAGGTGCAGTGCG  R：AGTGGTGCGTTGCTCTCTCT | 59 | 230 | 35,423 | 93.5 |
| si296 | 1 | F：AGAACATGCATGGAGGAACC  R：ACATGAGAAAGTTTCGCGCT | 59 | 192 | 34,358 | 96.1 |
| GA4 | 1 | F：ATGCAACGAAAGGTCTCAAACTAC  R：GTGTGTTTCCCTTTGCCAATGATA | 55 | 190 | 37,689 | 123.3 |
| p8 | 1 | F：CGATCGAATGATCGATGAAC  R：CCCTTTGTCCGATCACGTC | 55 | 203 | 37,804 | 124.0 |
| b260 | 1 | F：GAAGAGAGAAGCAGCGTTC  R：AAACCACACTTGCCCTGA | 55 | 159 | 37,750 | 124.0 |
| b249 | 2 | F：ACCTGGTCTTCGTTTTGG  R：AAAATTCTGCACCCAATGAA | 47 | 131 | 46,087 | 0.0 |
| p80 | 2 | F：GCCGTTGGATTTGATTATGG  R：TGTGGTTAGTTTATGTGGCTTG | 55 | 202 | 44,317 | 4.3 |
| GC35 | 2 | F：CTTGGGACTGAAAGGCTATG  R：ATGCGTTTGGTGGTTTTG | 55 |  | 44,015 | 5.8 |
| p56 | 2 | F：GATGTGTACGGGTTGCATTG  R：TGGGTTTCAGGGCTCTCTC | 55 | 199 | 41,256 | 20.5 |
| p39 | 2 | F：GAACACAAAACCTGGGAACG  R：TTCGCAGTAAACGTGCAAAC | 57 | 195 | 39,338 | 34.0 |
| SIMS5318 | 2 | F: TCCTTGGTAGCAGCACCTCT  R: GGTGAAGGATGGCAATGACT | 60 | 228 | 37,770 | 37.5 |
| GD13 | 2 | F：ATGTCAGCATGAGTAACATTCTCG  R：TTGGTGATGGATTCGTCATTAG | 55 | 184 | 27,259 | 62.9 |
| b157 | 2 | F：ATTCGTTTGTGTTCTGGCAAT  R：GTGTCTCTTGCCCTCGTTTC | 57 | 222 | 6,524 | 79.7 |
| si141 | 2 | F：CTCTCCGCACTACTCATCCC  R：CAGAAAGTCCACTGGTTGCC | 59 | 105 | 4,383 | 87.2 |
| SIMS3715 | 2 | F: ACCCAGATGGGTGACAACTC  R: GCTGCAACTGTGATGGAAGA | 60 | 289 | 3,028 | 103.1 |
| si036 | 2 | F：GCCTGTGCACTGCTAACAAA  R：TACAGTGCATGGGTGAAAGG | 59 | 103 | 1,228 | 133.0 |
| si109 |  | F：AGCAAGACAGAGAAGAGCGG  R：GAGAGTGGAGGAATGGACGA | 59 | 276 | Unknown | 143.8 |
| b198 | 3 | F：AGGGGACATGATCTCCAAAG  R：CTGCCCAAAGTTGAGCTGTT | 59 | 242 | 48,184 | 0.0 |
| b163 | 3 | F：CTCGGAAGCTCAGATTCTCC  R：CACTTCCTGCAGCTCTCACA | 57 | 194 | 47,639 | 18.7 |
| si093 | 3 | F：CTCGTGTGTTTGGAGCAAGA  R：TCGGTGCATCTCTGAACAAG | 59 | 114 | 39,736 | 40.1 |
| p78 | 3 | F：CGCCCCTGTGTTACCATTC  R：CGTTGTCGAAAACCATTCAAG | 57 | 203 | 23,125 | 45.7 |
| si273 | 3 | F：CCACCTGAGCCTACTCTTGC  R：AGATGCCTGCAGCAAATTCT | 59 | 220 | 18,976 | 55.0 |
| p61 | 3 | F：CATCCGCGTCATCTGAATC  R：ACCTGCTGCTATCCATCACC | 56 | 200 | 18,478 | 56.2 |
| b186 | 3 | F：CCCGTATAAATGTCATCATCCC  R：GCACCTGGCTTCCCTTT | 55 | 191 | 17,566 | 57.8 |
| p98 | 3 | F：ATTCATCAGTAGCACAGC  R：TGGAACTAAGAACAGGAAAC | 55 | 155 | 15,515 | 58.1 |
| b225 | 3 | F：ACCAAGAACTGCCTGCAC  R：TGCTTAGAACCCACTTGATCG | 55 | 131 | 11,756 | 61.8 |
| b114 | 3 | F：TTCTGTCCTTTCGGGAGATG  R：CTCGTCGTCACTACACATGGA | 54 | 180 | 5,764 | 77.5 |
| GA49 |  | F：TCTTGCTCCTATTCTGTCCTT  R：AACCATTCTCGTCAATCACTAC | 55 | 231 | Unknown | 77.6 |
| si204 | 3 | F：CACTCGCAGGGACAACAGTA  R：CCAATGGGATGATGTTAGGG | 59 | 183 | 4,621 | 86.5 |
| si192 | 3 | F：TGCAACTGCAATCCCATTTA  R：CCAGCTTGAACAGGTTCTCC | 59 | 333 | 4,377 | 88.9 |
| GD5 | 3 | F：GAGCGAAAACTATCAGGTAACAGG  R：TTGCTAGGTAGCACAAGTTATTGC | 55 | 207 | 1,827 | 129.7 |
| CAAS3057 | 3 | F: ACACACATGCCCACCACCACCA  R: GCCATTGCCACATCTCTGTGCTCTC | 59 | 196 | 2,659 | 136.0 |
| si320 | 3 | F：GTGGGCAGAGACAGAGGAAG  R：CATACACACACACATGCCCA | 59 | 285 | 2,659 | 136.8 |
| p2 | 4 | F：GCCGAAACCCTTGTCTCTAC  R：CGCCACCAGCAACAATATC | 57 | 183 | 1,221 | 0.0 |
| b109 | 4 | F：TGTAGAGTGGCTAGGACCAT  R：GTTTCTTCCATCATGCCTTCTT | 56 | 175 | 1,290 | 3.4 |
| b113 |  | F：GTCCTTGGAACATCATT  R：TGAACACACTTACACTG | 43 | 186 | Unknown | 29.0 |
| b147 | 4 | F：CTACTGCCTTCTGGCCTCC  R：GGGCATTCTTGCTTCAGTCA | 57 | 216 | 8,777 | 54.0 |
| b255 | 4 | F：GAGGACAGCGGCCATT  R：CCTCCCTCCATTTACTTTGG | 55 | 195 | 6,833 | 57.0 |
| p89 | 4 | F：GCCTGTCTGAAAATTCTCAATG  R：AGACGTGACATTAGCGCTTG | 57 | 218 | 30,398 | 63.9 |
| p42 | 4 | F：GCGACTTTCCCCTTCCAATC  R：TTCCTTTTGTTGGCTTCTCC | 57 | 188 | 34,506 | 77.6 |
| b236 | 4 | F：TCTGGACCAGCATTCTGTCTT  R：GGTAACTCTGCTTGGACGAG | 55 | 156 | 37,687 | 98.9 |
| b210 | 4 | F：ACTCCTATCCAAGACCCAATCT  R：ACAGCCAACCAACCGGC | 55 | 192 | 38,759 | 116.9 |
| b247 | 4 | F：GATTGCTCTCTCACACACACG  R：GCCCGATGGCTGCTAGT | 55 | 116 | 39,395 | 125.9 |
| si324 | 4 | F：TGACGGATGGATGCTCATAA  R：CTCAGCTAACACCTACGGGC | 59 | 347 | 39,460 | 127.8 |
| GD46 | 4 | F：GATGGGTCGTCGTTAGAGTTT  R：GGAAAGGGAAAGGAAGATAGC | 55 | 124 | 39,463 | 127.8 |
| si350 | 4 | F：TGGCAGGCTCTCTCTCTCTC  R：GTACTTGCTGGCCTTTGCTC | 59 | 256 | 39,343 | 137.8 |
| b266 | 4 | F：AAGGAACAGATGGAGGCAT  R：AACACAAGACCACCAGAGA | 55 | 245 | 40,054 | 156.3 |
| b103 | 5 | F：CTACACCTCGCTTCCAGTCC  R：CTTCCATTTGCAGGATTGCT | 57 | 202 | 4,976 | 0.0 |
| b264 | 5 | F：GCGAATTCAGCTTAAACAGTTC  R：AACGGCTCTTTATCCGTGTA | 55 | 145 | 4,976 | 17.8 |
| SIMS6419 | 5 | F：GTGATGGGTCAGGATTTGCT  R：CAGCTCAGCTCAGTCAATCG | 59 | 210 | 9,671 | 42.4 |
| b158 | 5 | F：GATGAGGAAAAGGTAGGTTGGA  R：CTGCAACGTGCAGAACTACG | 57 | 206 | 25,188 | 57.6 |
| SIMS7613 | 5 | F：ACCTTCATTGACGCCTCATC  R：GCAGCAGCAGCAAATACAAA | 59 | 252 | 27,979 | 65.7 |
| CAAS5058 | 5 | F: AATGCGAGGAGGAGGAGCGGAAGA  R: AAATCGAATGGACGCGGACACCG | 59 | 180 | 34,707 | 82.8 |
| si118 | 5 | F：GAACTCATGCCAAGATGCAA  R：TCCTTGTGAAACCTGGGAAG | 59 | 343 | 31,069 | 92.5 |
| CAAS5027 | 5 | F: TCACGACGACGGTGAGCATTTTGC  R: TGATGCGCCGCCTGGTTCTTCT | 59 | 235 | 32,298 | 94.5 |
| SIMS7912 | 5 | F:ACCACATCTCCTATCCGCAC  R: GGAGATCGCAACAACCATCT | 60 | 146 | 34,022 | 95.8 |
| SIMS7821 | 5 | F:GAAGGGAGAGGAGGGCTAGA  R: AATTCCAAACACTGCAACCC | 60 | 181 | 32,372 | 97.7 |
| CAAS5034 | 5 | F: GTGTCCTCGCTCTCCACCAAGACTC  R:ACGAGACGTCAAATCCTGCAAATGGC | 59 | 150 | 37,301 | 108.5 |
| si017 | 5 | F：CCGTCGTGCTAGGATACGTT  R：AAGGTTGGTAGTTGCCCTTG | 59 | 298 | 30,646 | 145.3 |
| p17x | 5 | F：CGGACACCTGAAAGACGAA  R：GTCACTTGTTGTTGTTGCG | 55 | 213 | 43,358 | 205.7 |
| GA54 | 5 | F：GCGTGTGTGTAAAGCACGTAC  R：GGACACCTGAAAGACGAACAC | 55 | 240 | 43,358 | 207.6 |
| b234 | 6 | F：GCCGCAACGAACAACCG  R：CCTGTCCCTATCCCTGTCG | 55 | 290 | 25,797 | 0.0 |
| P10 | 6 | F：CAATCACATCCGAGCATTTC  R：CACCCACCGTGTTGATCTG | 55 | 198 | 7,486 | 7.9 |
| p32 | 6 | F：TTCAGGGATACACGAGCATTAC  R：CATCCATCCATGATCACCAG | 55 | 192 | 5,086 | 16.1 |
| b159 | 6 | F：GCCAGTCCGAGATGGTTAAG  R：AGCTCTAGCAGTTGGGGACA | 57 | 185 | 5,213 | 17.1 |
| si053 | 6 | F：CCGTTGGCAAGTACATGTTG  R：GCGTGTGTGTGTGTGTGTGT | 59 | 317 | 4,088 | 22.1 |
| p12x | 6 | F：ACGAGTCACAAATCACAGCAC  R：ATGCCTGAGCGGAACGGAA | 59 | 224 | 4,088 | 22.2 |
| si052 | 6 | F：CGCCATATATAGCAGCAGCA  R：AATCAAAGACGACGTGGGAG | 59 | 127 | 4,088 | 22.2 |
| si054 | 6 | F：CCGGTTTCTGTACGTGTGTG  R：GCGTACCGAGATGGAATGAT | 59 | 252 | 4,088 | 22.3 |
| GA5 | 6 | F：CACGAGAACATGGAAACAATTAGC  R：TATTGTCCCCCTACGTTCCTTGAG | 55 | 154 | 3,640 | 28.4 |
| si022 | 6 | F：ACCGGAGACATTGAGGTGAC  R：CTAGCACAAGACGTCGTCCA | 59 | 310 | 2,184 | 37.6 |
| si023 | 6 | F：CAAGCATGAACCATGTCCAC  R：TCCTGTATATGCACGGACCA | 59 | 281 | 2,183 | 39.7 |
| si100 | 7 | F：GGGTACGGTTGTCGATGTCT  R：AGCTTAGCCCAACAACCAGA | 59 | 361 | 2,752 | 0.0 |
| si265 | 7 | F：CACTGTCACCTTCAGACGGA  R：ATATCAAACTGGCTGGTGGC | 59 | 196 | 2,227 | 2.6 |
| si063 | 7 | F：GACCAAGCAGCTGTAAACTCG  R：TCTTGATCATGCAGGGCATA | 59 | 255 | 2,232 | 2.8 |
| GA12 | 7 | F：ACTTGCCAAGGAACCACCCAAAGC  R：GCTAGGCATTGTGCTCACGTAAAG | 55 | 257 | 3,631 | 7.6 |
| CAAS7003 | 7 | F: CATCATTGCACGCATGTTC  R: TCAACTTTGTCCTAAGAGAGACG | 55 | 138 | 12,704 | 12.4 |
| CAAS7009 | 7 | F: GGTCACAGCCAAGTGTTTTGTC  R: TCAACTACAGGAATCCCAACCA | 58 | 270 | 17,637 | 36.5 |
| b142 | 7 | F：TGGTAAAACTCCCATATTGAGC  R：GCCCCATCCTTGATAACAGA | 57 | 232 | 17,691 | 40.4 |
| SIMS1385 | 7 | F：GCGAGCTATACCTCTCACCG  R：TGATGGAGCAGCAAGAAATG | 59 | 183 | 18,296 | 44.6 |
| b192 | 7 | F：CACAATTCTCACCACGCCT  R：ACTATTCAGCTCGCCGTCA | 57 | 115 | 18,782 | 49.2 |
| SIMS1394 | 7 | F：CCTGGATAGGATTGCTTGGA  R：GCATGCAGTTGCGAGTTAAA | 59 | 235 | 19,397 | 52.2 |
| si227 | 7 | F：GCGAGCTATACCTCTCACCG  R：TGATGGAGCAGCAAGAAATG | 59 | 183 | 18,296 | 68.2 |
| p45 | 7 | F：CATGCATCGTGAGGGAATC  R：AATTGCTCCCATGCTACTGTG | 57 | 192 | 33,940 | 123.3 |
| SIMS1712 | 7 | F: AGCGTACGAGGACCTCAAGA  R: TTAGATCGGTGTCGGTGTCA | 60 | 158 | 33,480 | 131.5 |
| si178 | 7 | F：ATCATGCGCAAACATTACGA  R：AATTGGAGCATGTACCGGAG | 59 | 135 | 33,319 | 131.6 |
| si136 | 7 | F：ACTTCGTCATTGCCTCCTTG  R：CTAACAAATTTGGGCGGAGA | 59 | 133 | 33,221 | 137.1 |
| si256 | 7 | F：CTCCAAGCATCTGTCCCATT  R：TCAGCTTGTACTTGTTGCCG | 59 | 384 | 30,571 | 160.7 |
| si119 | 7 | F：GCTGAGAAAGTTTGTTGGGC  R：GAATTCGAACCGAGCACATT | 59 | 293 | 27,196 | 178.8 |
| p59 |  | F：TAATTTTGTGGCGTGGGATG  R：GCACTGGTTTTGTTGAATGG | 57 | 176 | Unknown | 180.6 |
| SIMS1445 | 7 | F: GGCAGTTCCGCTACATCCTA  R: CTTGGAGAGCTTGATGAGGG | 60 | 132 | 26,663 | 183.4 |
| SIMS1409 | 7 | F：TCTTTCACGGTGCTTTAGGG  R：GAGAGTAGCTCGAGCGGAAA | 59 | 359 | 21,648 | 198.6 |
| GC33 | 7 | F：TTGGGTTTCAACTCTAGCCTAC  R：GATCCTGAAGCATTCGATAAAC | 55 |  | 23,661 | 208.1 |
| b185 |  | F：GCACGTGTGACTTTCCACAT  R：GTGAATGGCACACGAAACTG | 55 | 167 | Unknown | 0.0 |
| SIMS1239 | 8 | F: CCATGCTTACACGGAATGTG  R: CCTCCGCTCCAAACAAATAG | 60 | 274 | 27,626 | 29.3 |
| si358 | 8 | F：CAACAAGGTTGGTTGGCTTT  R：CAGACACAGAGCAACAGGGA | 59 | 220 | 8,825 | 33.2 |
| p14 | 8 | F：TCGTTCAGGCTCAGGACATC  R：GAACAAGAAAGAACATCCTGTGG | 59 | 210 | 10,551 | 33.2 |
| si318 |  | F：GTTTGTGACGAGGGATTCGT  R：CGGAATCACCGCTTATTGAT | 59 | 192 | UnKnown | 35.3 |
| SIMS1170 | 8 | F：GTTACAAAGACATGGGCGCT  R：TGCTCGATGTCGAAACAAAG | 59 | 359 | 7,443 | 40.3 |
| p6 | 8 | F：AAGGATGGAATTTGCCACTG  R：TTTCGACGATTTGCTTCAAC | 58 | 188 | 7,222 | 40.3 |
| SIMS1161 | 8 | F：AATTCCTCCCTCCAACATCC  R：CAAGAAGCAGCTACCGAACC | 59 | 157 | 5,924 | 45.6 |
| b222 | 8 | F：GGCATGCATGGAACAAAA  R：AGACGAGCTGAACTTGAGG | 50 | 131 | 26,040 | 58.7 |
| SIMS1275 | 8 | F: CTCCAACGGTGTTCCAACTT  R: TCCCACCACTGTAGACACCA | 60 | 162 | 34,492 | 93.3 |
| b258 | 8 | F：GGGCCAATAATGGTTGCATA  R：TTGCACATCCAAATCTTTCC | 55 | 267 | 35,174 | 95.5 |
| si172 |  | F：CATATCATCCAACCCGCTTC  R：GAGTTAGGGTTCAGCTTGCG | 59 | 155 | Unknown | 108.0 |
| CAAS9058 | 9 | F: ACACGGTCAGTCACGCAGGTCT  R: CAGCTGCAGTGGTGGAGGAACG | 59 | 115 | 44,003 | 0.0 |
| b174 | 9 | F：TTTCGGGTAAGAATTGAGATGG  R：GGTAGCAAGGTGACAAAGTT | 55 | 168 | 46,820 | 13.3 |
| p41 | 9 | F：CGTGCGTTATGTGATCCTAGC  R：ACGTTTGCCTCTGCTTCTTG | 59 | 202 | 51,081 | 22.2 |
| b105 | 9 | F：ACTTGCATTGGTCGCCTTTA  R：ACGCGCATTCAATCAGACTA | 57 | 199 | 52,908 | 28.0 |
| b145 | 9 | F：CGCCTCTATGGGTCTAACA  R：AGGTTTCTTTGGTCCGTTTC | 54 | 102 | 56,494 | 36.6 |
| b166 | 9 | F：CGCCCATACTACCCAACAG  R：ACCTCACCTTCCACTCCTC | 57 | 198 | 14,109 | 69.1 |
| b217 | 9 | F：TGCAGCAGCTAGGGAGG  R：CCGAATGCACGGTGATGA | 55 | 286 | 11,197 | 84.5 |
| b246 | 9 | F：CACGCACGTAGTATTGCTAT  R：GTTCTGGGCTTCTGGCTG | 55 | 128 | 6,681 | 115.2 |
| si177 | 9 | F：CATCGCTCCAATTCCAATCT  R：TAGCTGCTCGAAGCTCAACA | 59 | 204 | 6,786 | 123.3 |
| CAAS9018 | 9 | F: TGCTTTGCATTGGACGACCAGAGA  R: GCCGGAGGCTTCTTCCGACAG | 57 | 131 | 3,852 | 154.5 |
| p44 | 9 | F：TTCCCGGAACAGACAAGAAC  R：GCGTTGGAAGCCATGGAG | 59 | 190 | 3,452 | 169.6 |

**Table S1** Physical positions and primers sequences of SSR markers used in this trial
