# Supplementary material for: Mapping of Quantitative Trait Locus (QTLs) that Contribute to Germination and Early Seedling Drought Tolerance in the Interspecific Cross Setaria italica×Setaria viridis
Source: PLoS One. 2014 Jul 17;9(7):e101868. doi: 10.1371/journal.pone.0101868 (PMC4102488; doi:10.1371/journal.pone.0101868)
Supplement: Table S2 — SSR markers showing segregation distortion (P<0.01) in the Yugu1/W53 F7 RIL population and their co-localizations with segregating skewness regions annotated in other populations. (DOC) [file pone.0101868.s004.doc]

**Table S2.** SSR markers showing segregation distortion (P<0.01) in the Yugu1/W53 F_7_ RIL population and their co-localizations with segregating skewness regions annotated in other populations

| **SSR Marker** | **Chromosome** | **Number of observed alleles** | | **Direction of skewness** | **χ^2^ value** | ***P^a^* value** | ***P^b^* value** | ***P^c^* value** |
| --- | --- | --- | --- | --- | --- | --- | --- | --- |
|  |  | **Yugu1** | **W53** |  |  |  |  |  |
| P52 | 1 | 107 | 67 | Yugu1 | 9.195 | 0.002427 | 0.884027 | 0.28505 |
| GA4 | 1 | 101 | 60 | Yugu1 | 10.441 | 0.001232 | 0.714636 | 0.20325 |
| Si141 | 2 | 111 | 61 | Yugu1 | 15.211 | *9.61E-05* | *4.63E-11* | 0.46533 |
| P56 | 2 | 109 | 64 | Yugu1 | 11.71 | *0.000622* | *0.001254* | 0.41204 |
| P39 | 2 | 107 | 61 | Yugu1 | 12.595 | *0.000387* | *3.91E-07* | 0.52034 |
| GD13 | 2 | 60 | 113 | W53 | 16.24 | *5.58E-05* | *1.01E-24* | 0.32368 |
| Si192 | 3 | 129 | 59 | Yugu1 | 26.064 | 3.3E-07 | 0.213807 | 0.63773 |
| Si204 | 3 | 115 | 62 | Yugu1 | 15.87 | 6.78E-05 | 0.341779 | 0.63773 |
| Si273 | 3 | 122 | 44 | Yugu1 | 36.651 | 1.41E-09 | 0.106718 | 0.53036 |
| Si093 | 3 | 114 | 61 | Yugu1 | 16.051 | *6.17E-05* | *0.026992* | 0.25112 |
| b186 | 3 | 115 | 50 | Yugu1 | 25.606 | 4.19E-07 | 0.056596 | 0.52543 |
| P78 | 3 | 114 | 61 | Yugu1 | 16.051 | *6.17E-05* | *0.018958* | 0.57303 |
| b225 | 3 | 115 | 50 | Yugu1 | 25.606 | *4.19E-07* | *0.001479* | 0.51329 |
| b163 | 3 | 115 | 49 | Yugu1 | 26.561 | 2.55E-07 | 0.557480 | - |
| GD5 | 3 | 110 | 51 | Yugu1 | 21.621 | 3.32E-06 | 0.122600 | 0.15038 |
| Si324 | 4 | 107 | 67 | Yugu1 | 9.915 | *0.001639* | *9.13E-19* | - |
| b210 | 4 | 105 | 64 | Yugu1 | 9.947 | *0.001611* | *2.66E-17* | - |
| b236 | 4 | 117 | 59 | Yugu1 | 19.114 | *1.23E-05* | *1.61E-14* | - |
| b109 | 4 | 105 | 59 | Yugu1 | 12.902 | 0.000328 | 0.510444 | 0.84353 |
| b255 | 4 | 117 | 46 | Yugu1 | 30.926 | *2.68E-08* | *0.048332* | 0.11385 |
| SIMS6419 | 5 | 120 | 66 | Yugu1 | 15.677 | *7.51E-05* | *0.003357* | 0.17434 |
| SIMS7912 | 5 | 109 | 62 | Yugu1 | 35.083 | *3.16E-09* | *0.000386* | *0.01364* |
| GA54 | 5 | 52 | 110 | W53 | 20.765 | *5.19E-06* | *1.78E-07* | - |
| b103 | 5 | 46 | 131 | W53 | 40.819 | *1.67E-10* | *0.000339* | *0.00041* |
| Si023 | 6 | 110 | 61 | Yugu1 | 14.041 | *0.000179* | *0.000137* | *0.00944* |
| b234 | 6 | 105 | 64 | Yugu1 | 9.947 | *0.001611* | *4.51E-07* | 0.89225 |
| b159 | 6 | 125 | 52 | Yugu1 | 30.107 | *4.09E-08* | *3.62E-06* | 0.06298 |
| GA5 | 6 | 116 | 48 | Yugu1 | 28.195 | *1.1E-07* | *0.000386* | 0.19295 |
| SIMS1409 | 7 | 65 | 119 | W53 | 15.848 | 6.86E-05 | 0.826352 | 0.32157 |
| SIMS1170 | 8 | 44 | 123 | W53 | 37.371 | 9.77E-10 | 0.941072 | 0.32988 |
| b222 | 8 | 142 | 34 | Yugu1 | 66.273 | 3.93E-16 | 0.186893 | 0.73429 |
| P14 | 8 | 106 | 60 | Yugu1 | 12.747 | *0.000357* | *0.001254* | 0.27149 |
| b217 | 9 | 117 | 59 | Yugu1 | 19.114 | 1.23E-05 | 0.106227 | 0.25281 |
| P41 | 9 | 106 | 60 | Yugu1 | 12.747 | *0.000357* | *0.005331* | - |
| b166 | 9 | 61 | 110 | W53 | 14.041 | 0.000179 | 0.164705 | 0.89685 |

a: This study; b: B100×A10 RIL population [24]; c: Zhanggu×A2 F2 population [34]
